# Predictive value of cardiac magnetic resonance mechanical parameters for myocardial fibrosis in hypertrophic cardiomyopathy with preserved left ventricular ejection fraction

**DOI:** 10.3389/fcvm.2022.1062258

**Published:** 2022-12-15

**Authors:** Xian Hou, Xing Xiong, Xia Li, Jianhua Bi, Gaofeng Xu, Yining Wang, Shu Jiang

**Affiliations:** ^1^Department of Radiology, Quzhou Kecheng People’s Hospital, Quzhou, China; ^2^Department of Radiology, The First Affiliated Hospital of Soochow University, Suzhou, China; ^3^Department of General Medicine, The Sixth Affiliated Hospital of Nantong University, Yancheng Third People’s Hospital, Yancheng, China; ^4^Department of Medical College, Jiangsu Vocational College of Medicine, Yancheng, China; ^5^Department of Radiology, The First people’s Hospital of Yancheng, The Yancheng Clinical College of Xuzhou Medical University, Yancheng, China; ^6^Department of Radiology, Peking Union Medical College, Chinese Academy of Medical Sciences, Peking Union Medical College Hospital, Beijing, China

**Keywords:** hypertrophic cardiomyopathy, myocardial contraction fraction, late gadolinium enhancement, cardiac magnetic resonance, myocardial fibrosis, myocardial strain

## Abstract

**Objective:**

Myocardial fibrosis leads to systolic dysfunction in hypertrophic cardiomyopathy (HCM) patients. This study aims to investigate the relationship between cardiac magnetic resonance mechanical parameters for evaluating the left ventricular function in HCM with preserved left ventricular ejection fraction (LVEF ≥50%) and the association between myocardial fibrosis defined by late gadolinium enhancement (LGE).

**Methods:**

This study was a retrospective analysis of CMR images of 93 patients with HCM with preserved ejection fraction (HCMpEF) and 96 controls diagnosed by cardiac magnetic resonance (CMR) at our hospital from July 2019 to January 2022. The myocardial contraction fraction (MCF) was calculated, and myocardial mechanical parameters, including global myocardial longitudinal strain (GLS), circumferential strain (GLS), and myocardial strain (GLS), were obtained by tissue tracking and LGE quantitative modules of dedicated software, respectively. The correlation between myocardial strain and LGE was analyzed, and a multivariate logistic regression model was developed to discuss the risk predictors of LGE.

**Results:**

Compared to the control group, the left ventricular mechanical parameters GLS (−13.90 ± 3.80% versus −18.20 ± 2.10%, *p* < 0.001), GCS (−16.62 ± 3.50% versus −18.4 ± 2.69%, *p* < 0.001), GRS (28.99 ± 10.38% versus 33.02 ± 6.25%, *p* < 0.01), and MCF (64 ± 16% versus 99 ± 18%, *p* < 0.001) were found significantly lower in HCM group. Moreover, even in LGE-negative HCM patients, GLS (−16.3 ± 3.9%) and MCF (78 ± 19%) were significantly lower compared to the control group. Left ventricular GLS [OR = 1.61, (1.29, 2.02), *p* = 0.001] and MCF [OR = 0.90, (0.86, 0.94), *p* = 0.001] independently predicted myocardial late gadolinium enhancement (LGE).

**Conclusion:**

In participants of HCM with preserved ejection fraction, the early onset of reduced left ventricular GLS and MCF in patients with HCMpEF may provide new evidence for evaluating impaired myocardial systolic function. The reduction of myocardial mechanical indexes may reflect the presence and extent of myocardial fibrosis, and the more significant the reduction, the more severe the myocardial fibrosis; GLS and MCF may be ideal predictors for LGE.

## Introduction

Hypertrophic cardiomyopathy (HCM) is common genetic cardiomyopathy and the leading cause of sudden death in adolescents and athletes. The clinical manifestations of HCM are highly variable, and patients may be asymptomatic for a long time with a good prognosis, but some patients may experience major cardiovascular adverse events (MACEs) such as arrhythmia, heart failure, and sudden death (1). Myocardial fibrosis plays an important role in the pathophysiological mechanisms of myocardial injury dysfunction and is an important cause of sudden cardiac death and heart failure in patients with HCM (2). Endomyocardial myocardial biopsy is the gold standard for diagnosing myocardial fibrosis, but its invasive nature and complications sometimes limit its clinical application. Cardiac magnetic resonance (CMR) examinations are increasingly used to follow up and assess changes in left ventricular hypertrophy (LVH) and myocardial fibrosis over time, which are prognostic variables in HCM. Myocardial late gadolinium enhancement (LGE) is a well-established technique and is considered the “gold standard” for myocardial fibrosis assessment because it can accurately assess the extent and degree of myocardial fibrosis (3, 4). However, gadolinium administration can cause nephrogenic systemic sclerosis in patients with impaired renal function. In addition, gadolinium may be deposited in the skin, liver, and brain (5). There is still controversy surrounding the implications of gadolinium accumulation in different organs, so rationalizing the use of gadolinium in CMR evaluation of HCM patients, who are often young and need repeated CMR scans, would be beneficial. Moreover, the extent of systolic dysfunction caused by myocardial fibrosis can be challenging to determine on cardiac MRI, despite the ability to assess LGE for myocardial fibrosis (6). Previously, our group studied myocardial involvement in autoimmune diseases such as SLE based on mechanical parameters such as myocardial stress from cardiac ultrasound speckle tracking technique and found that metrics of myocardial mechanics can assess myocardial systolic dysfunction, timely detect myocardial damage, and are predictive of myocardial fibrosis (7). CMR has higher reproducibility and spatial and temporal resolution than cardiac ultrasound and is the gold standard for assessing cardiac structure and function (8). Therefore, if mechanical indices can be obtained based on contrast-free CMR sequences, and the presence and myocardial fibrosis can be predicted without or before the application of LGE, early clinical therapeutic decisions can be making in addition to shorten the examination time. Previously, CMR-feature tracking (CMR-FT) analysis had also been used to assess myocardial fibrosis (9) which can provide data on LV strain, but is not routinely available on standard PACS and requires additional software.

Myocardial contraction fraction (MCF), based on CMR images, is a quantitative mechanical parameter derived from each standard CMR examination without requiring specialized post-processing software. In this study, in addition to the LV strain provided by CMR-FT, we also aim to evaluate the MCF, analyze the correlation between these myocardial mechanical indices and LGE, and further investigate whether these non-enhanced cine images from hypertrophic cardiomyopathy patients with preserved ejection fraction (HCMpEF) could be used to predict the presence of myocardial fibrosis.

## Materials and methods

### Patients

**FIGURE 1:**
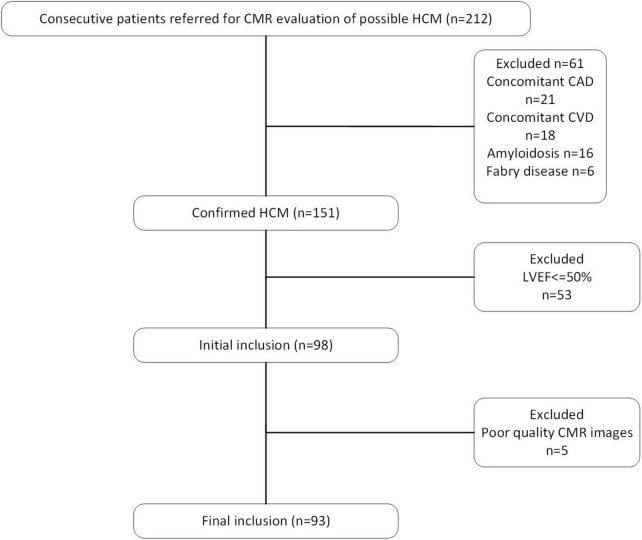
The flowchart of inclusion and exclusion criteria.

In this retrospective observational study, consecutive adult patients (>18 years old) referred to clinical CMR were analyzed from a prospectively maintained clinical CMR database and a retrospective review of all patients referred for HCM between 2019 and 2022 who underwent CMR was performed. In total, 151 patients met the diagnostic criteria. The diagnostic criteria of HCM (10) mainly included unexplained septal hypertrophy with a maximal left ventricular wall thickness of 15 millimeters (mm). The exclusion criteria were a history of chemical ablation and surgery before CMR examination and left ventricular ejection fraction <50%. The patients of HCMpEF enrollment procedure are shown in Figure 1.

In addition, 96 cases (45 males) with normal CMR imaging results, who did not exhibit cardiovascular disease or diabetes symptoms, including cardiac surgery or interventions, were enrolled in this study.

### Cardiac MRI protocol

A SIMENS 3.0T (MAGNETOM Skyra, Siemens Healthcare, Erlangen, Germany) magnetic resonance scanner was used to perform the standard cardiac magnetic resonance imaging examination. The data were acquired using an 18-channel body matrix coil and a 32-channel spine array coil using an MR-compatible wireless vector 4-lead vector cardiac gating board. Cine images were acquired by cardiac gated 2D steady-state free-feed sequence (SSFP) acquisition after multiple breath-holding, including 2-, 3-, and 4-chamber long-axis and 10–12-layer ventricular short-axis two-chamber images covering the entire left ventricular base to the apex; a 10-min delay after Gd-DTPA injection at a dose of 0.2 mmol/kg was used for cardiac gated breath-holding phase-sensitive inversion recovery (PSIR) sequence to obtain delayed enhanced images.

### CMR image post-processing

Cardiac magnetic resonance images were transferred to a Siemens workstation to automatically map the short-axis cine images of the left ventricle using Argus (SIMENS, Germany) software to determine morphological and functional parameters. The endocardial and epicardial contours were semi-automated generated, including the left ventricular end-diastolic volume (LVEDV), LV end-systolic volume (LVESV), LVEF, LV stroke volume (LVSV), and LV mass. MCF was obtained by the following formula: MCF = LV Stroke volume LV mass/1.05 × 100%.


(1) $$M\,C\,F=\frac{\text{EDV}-\text{ESV}}{\mathrm{LV}\,\mathrm{mass}/1.05}\times 100\%$$

Eq.1. The equation of MCF; MCF, myocardial contraction fraction.

### Myocardial strain parameters

**FIGURE 2:**
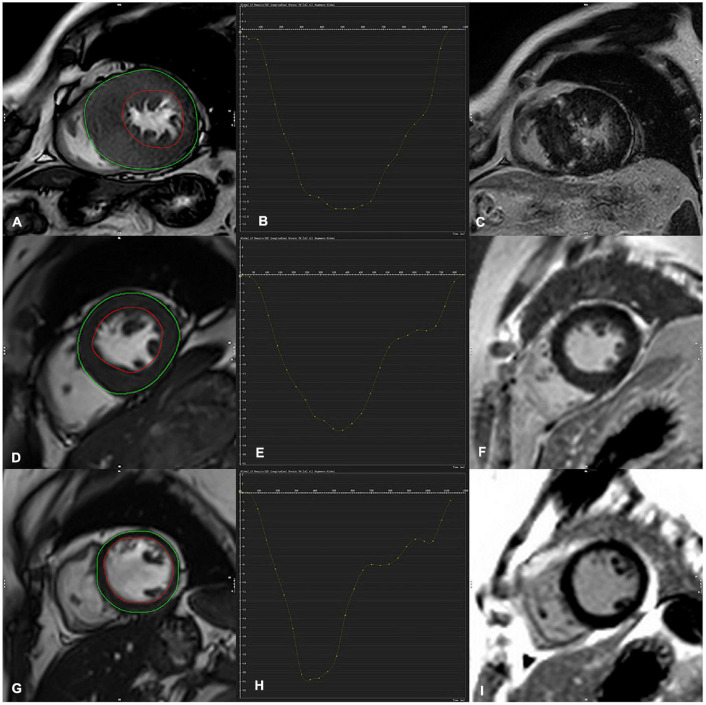
Contouring for left ventricular (LV) strain, global longitudinal strain (GLS) derived from cine images **(A,D,G)** (both endocardial and epicardial boundaries automatically delineated) **(C,F,J)** were corresponding LGE image. **(D–F)** Indicated a representative case of LV tracking in the short-axis view in a 36-year-old-male HCM patient with no LGE and a 52-year-old male HCM patient with LGE positive (upper row **A–C**), GLS in HCM LGE + patients significantly decreased in comparison with LGE- and a control subject (GLS = –12.0, –17.3, –21, respectively).

The myocardial strain parameters were obtained using the Canadian Cardiac Magnetic Resonance Professional analysis software. The tissue tracking module of the software Circle Cardiovascular (CVI42 Version 5.13.5) was used to analyze the cine images, and the software automatically performed deformation analysis to obtain 2D strain parameters, including global longitudinal 2D strain (GLS), global circumferential 2D strain (GCS), and global radial 2D strain (GRS) (Figure 2).

### Qualitative and quantitative LGE analysis

The late gadolinium enhancement (LGE) image evaluation procedure was performed by two blinded readers (one had 3 years of experience in cardiovascular imaging, while the senior radiologist had 6 years of experience). Patients were assigned into two subgroups based on whether they had visual LGE (LGE+) or not (LGE-). In case of differences in opinion, a consensus was reached by discussion. The endocardial and epicardial borders were manually drawn on the delayed reinforcement left ventricular two-chamber short-axis images using the LGE quantification module in CVI software. LGE myocardial volume was quantified by five standard deviations (SD) of the distal normal myocardial signal above the same level (11), and the software calculation automatically obtained LGE myocardial volume score.

### Statistical methods

SPSS 26.0 and GraphPad Prism version 8 software were used for data analysis, and measures that conformed to a normal distribution were expressed as $\bar{X}$ ± s (mean ± standard deviation), and those that did not were expressed as median (interquartile range). Comparisons between the two groups were performed using the independent samples *t*-test or Mann–Whitney U test. One-way ANOVA analysis of variance was performed to determine statistical significance between three groups, followed by the Bonferroni multiple comparison test. Univariate and multivariable linear regression analyses of LV myocardial mechanical parameters were used to assess how mechanical parameters correlated with the area of LGE using a stepwise selection method to identify variables that were correlated with the area of LGE by multifactorial regression analysis, where variable inflation factors (VIF) ≥ two were excluded for parameters considered to have significant collinearity. Further multivariate logistic regression analysis was performed, and the final analysis included independent variables modeled with logistic regression diagnostic models. The receiver operating characteristic (ROC) curves were plotted, and area under curves (AUC) were compared. Two-sided significance tests determined statistics with a *p* < 0.05 indicating statistical significance.

## Results

### Clinical characteristics of subjects

A total of 93 cases, including 57 males in HCM group and 96 cases, including 45 males in the control group, were included in this study without statistical difference in gender and age between the two groups. Among biochemical indexes, N-Terminal pro-brain natriuretic peptide (NT-proBNP), high–sensitive troponin T (hs-TnT), and creatine kinase (MB) were significantly higher in HCM group than in the control group (Table 1).

**TABLE 1:** Comparison of clinical and imaging parameters of the study population.

Parameter	Control (*n* = 96)	HCM patients (*n* = 93)	*P*
**Patients demographics**			
Age (year)	45 ± 14	51 ± 16	0.14
Male, *n* (%)	45 (47)	55 (59)	0.09
BMI (kg/m^2^)	23.46 (20.31, 23.57)	23.51 (22.32, 25.05)	0.64
**Biomarkers**			
NT-proBNP (pg/ml)	25.0 (17.0, 51.5)	152.5 (36.0, 392.3)	0.001
hs-TnT (pg/ml)	7.6 (5.7, 10.6)	13.4 (8.4, 30.9)	0.003
MB (ng/ml)	26.0 (21.5, 35.0)	27.3 (21.0, 40.8)	0.76
**Left ventricle variables**			
LVEDV/BSA (ml/m^2^)	66 ± 15	70 ± 17	0.53
LVESV/BSA (ml/m^2^)	27 ± 8	33 ± 14	0.56
LVEF (%)	59 ± 8	57 ± 7	0.17
LV mass/BSA (g/m^2^)	42 ± 8	78 ± 24	0.001
GLS (%)	−18.20 ± 2.10	−13.90 ± 3.80	0.001
GCS (%)	−18.4 ± 2.69	−16.62 ± 3.50	0.001
GRS (%)	33.02 ± 6.25	28.99 ± 10.38	0.002
MCF (%)	99 ± 18	64 ± 16	0.001
LGE area (%)	−	19.8 ± 14.3	–
LGE (+) [*n* (%)]	−	69(74)	–

HCM, hypertrophic cardiomyopathy; BMI, body mass index; LVEDV, left ventricular end-diastolic volume; LVESV, left ventricular end-systolic volume; LVEF, left ventricular ejection fraction; LVmass, left ventricular mass; LGE, late gadolinium enhancement; GLS, global myocardial longitudinal strain; GCS, global circumferential strain; GRS, global radial strain; MCF, myocardial contraction fraction. Values are given as mean ± standard deviation or as medians (quartiles 1-quartiles 3) for continuous variables and count (%) for categorical variables.

### Magnetic resonance structural and functional parameters

Global myocardial longitudinal strain, GCS, GRS, and MCF were significantly lower in HCM group compared with the control group. In contrast, the LV mass was significantly higher in HCM group than in the control group (Table 1).

### Mechanical parameters of left ventricle based on LGE subgroups

**FIGURE 3:**
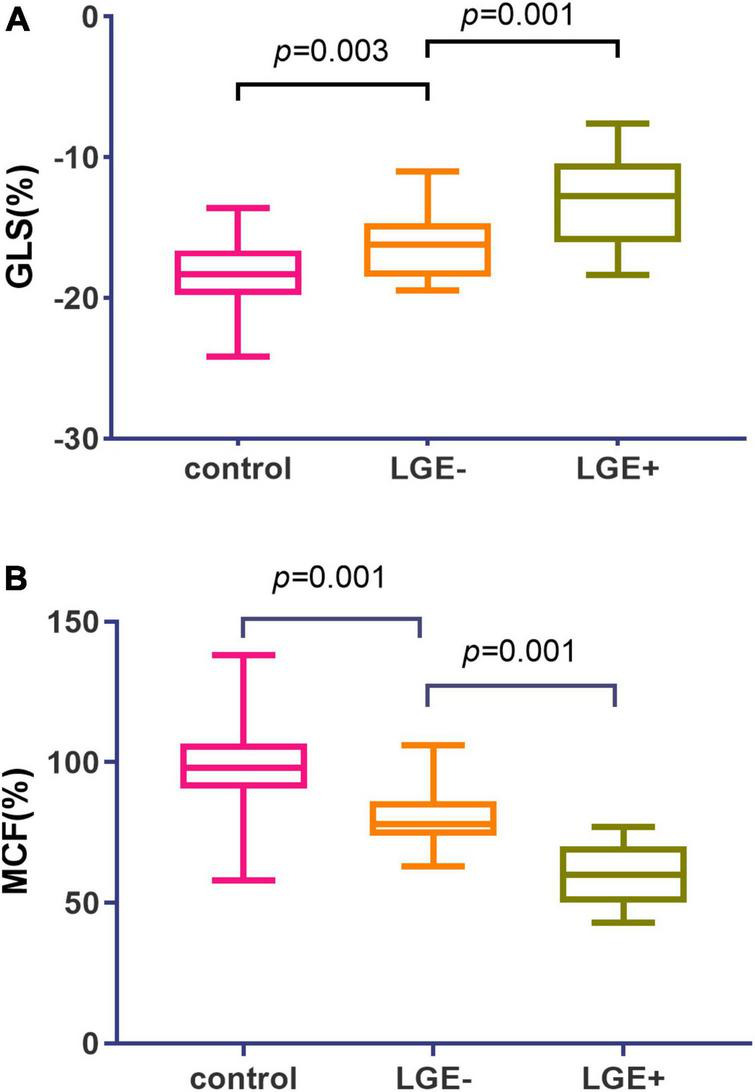
Global myocardial longitudinal strain (GLS) and myocardial contraction fraction (MCF) values in hypertrophic cardiomyopathy (HCM) subgroups with or without LGE. **(A)** Patients with LGE + exhibited a stepwise elevation in GLS (–13.1 vs. –16.3 vs. –18.2) as compared to LGE- subgroup and controls. **(B)** Patients with LGE + exhibited a stepwise reduction in MCF (98.6%vs. 78.5%vs. 59.3%) as compared to LGE- subgroup and controls.

Hypertrophic cardiomyopathy patients were assigned into LGE positive (i.e., HCM + , *n* = 69) and LGE negative (i.e., HCM-, *n* = 24) subgroups. In HCM patients, further subgroup analysis was performed with or without LGE, showing that LV GLS, GCS, GRS, and MCF were all significantly lower in LGE (+) group (Table 2). GLS and MCF were significantly lower in LGE (−) group compared to the control group (*p* = 0.028, *p* = 0.001) (Figure 3), but differences in GLS, GCS, and GRS between the two groups were not statistically significant (Table 2).

**TABLE 2:** Comparison of mechanical parameters in the LGE + and LGE- subgroups.

	Control *n* = 96	LGE (−) *n* = 24	LGE (+) *n* = 69	*P* *	*P* **
GLS (%)	-18.2 @ 2.1	−16.3 ± 3.9	−13.1 ± 3.2	0.001	0.028
GCS (%)	-18.4 @ 2.69	−18.3 + 3.9	−16.5 ± 2.4	0.042	
GRS (%)	33.02 @ 6.5	32.4 ± 9.5	27.3 ± 5.6	0.005	0.001
MCF (%)	99 @ 18	78 ± 19	59 ± 10	0.002	

Abbreviations as Table 1 *: LGE (+) group vs. LGE (−) group; **: LGE (−) group vs. control group. Values are given as mean ± standard deviation for continuous variables.

### Correlation between myocardial mechanical parameters and LGE

**FIGURE 4:**
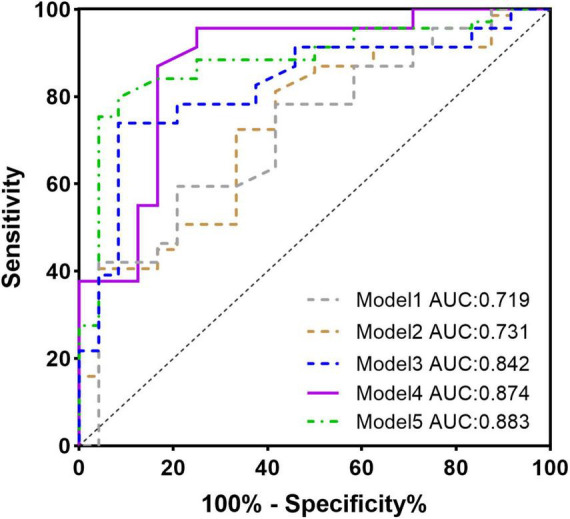
ROC curve for the multivariate logistic regression model predicting the presence of late gadolinium enhancement (LGE).

Univariate linear regression analysis revealed that MCF and myocardial strain parameters GLS, GCS, and GRS were significantly correlated with the area of LGE [MCF: β = −0.574, *R*^2^ = 0.43, *t* = −8.246, *p* < 0.001; GLS: β = 2.983, *R*^2^ = 0.657, *t* = −13.211, *p* < 0.001 13.211, *p* < 0.001; GCS: β = 1.30, *R*^2^ = 0.11, *t* = 3.237, *p* < 0.002; GRS: β = −0.313, *R*^2^ = 0.06, *t* = −2.248, *p* < 0.05]. In the multifactorial linear regression analysis corrected for age, gender, BMI, and LVEF, and after excluding significant collinearity with variance inflation factors (VIF) <2, GLS and MCF were still significantly associated with the area of LGE [β = 2.479, (2.066, 2.893) *R*^2^ = 0.74, *t* = 11.979, *p* < 0.001; β = −0.669, (−0.838, −0.501) *R*^2^ = 0.60, *t* = −9.867, *p* < 0.001]. LV mass was also significantly associated with the area of LGE [β = 0.193, (4.692, 6.833) *R*^2^ = 0.26, *t* = 4.017, *p* < 0.001]. The *t*-value shows the strength of the association, and *R*^2^ value expresses the extent to which the variables explain the variation in LGE area (Table 3). Five models were developed using logistic regression to predict LGE occurrence: model 1 included only the basal data, model 2 included LVEF, and models 3, 4, and 5 used LV_mass, MCF, and GLS instead of LVEF, respectively (Table 4). The model fit and predictive ability improved compared with model 1, with AUC increasing from 0.73 to 0.84, 0.88, and 0.87 (Figure 4). LV_mass [OR = 1.023 (1.001, 1.044) *p* = 0.037], MCF [OR = 0.90, (0.858, 0.944), *p* = 0.001], and GLS [OR = 1.613, (1.292, 2.015), *p* = 0.001] could independently predict LGE occurrence.

**TABLE 3:** Results of univariate and multivariate linear regression analysis of mechanical parameters and LGE.

Independent variable	Univariate β estimate (95% CI)	*P*-value	Multivariate β estimate (95% CI)	*P*-value
GLS	2.983(2.534,3.431)	0.001	2.479(2.066,2.893)	0.001
GCS	1.30(0.502,2.099)	0.002	0.353(−0.374,1.08)	0.335
GRS	−0.313(−0.59,0.036)	0.027	−0.036(−0.257,0.185)	0.42
MCF	−0.574(−0.712,0.436)	0.001	−0.669(−0.838,0.501)	0.001

CI, confidence interval. Other abbreviations as Table 1.

**TABLE 4:** Multivariate analysis (logistic regression) models showing odds ratio to predict the presence of LGE.

	OR (95% *CI)*	*P*-value
**Model 1**		
**Basic data:**		
Age (year)	1.034 (1.002−1.066)	0.035
Gender (male)	1.535 (0.508–4.640)	0.448
BMI (kg/m^2^)	1.269 (0.987–1.633)	0.064
**Model 2**		
**Basic data + LVEF:**		
Age (year)	1.034 (1.001–1.068)	0.042
Gender (male)	1.471 (0.457–4.729)	0.518
BMI (kg/m^2^)	1.350 (1.033–1.764)	0.028
LVEF (%)	0.946 (0.889–1.007)	0.081
**Model 3**		
**Basic data + LV_mass:**		
Age (year)	1.059 (1.020–1.100)	0.031
Gender (male)	1.501 (0.436–5.174)	0.520
BMI (kg/m^2^)	1.633 (1.200–2.223)	0.062
LV_mass (g/m2)	1.023 (1.001–1.044)	0.037
**Model 4:**		
**Basic data + GLS:**		
Age (year)	1.037 (0.999–1.077)	0.056
Gender (male)	1.228 (0.282–5.347)	0.784
BMI (kg/m^2^)	1.363 (0.979–1.898)	0.067
GLS (%)	1.613 (1.292–2.015)	0.001
**Model 5:**		
**Basic data + MCF**		
Age (year)	1.060 (1.015–1.107)	0.009
Gender (male)	1.463 (0.335–6.381)	0.613
BMI (kg/m^2^)	1.325 (0.948–1.852)	0.100
MCF (%)	0.900 (0.858–0.944)	0.001

OR, Odds ratio. Other abbreviations as Table 1.

## Discussion

This study showed that myocardial strain and MCF in all domains (longitudinal, radial, and circumferential) were significantly decreased in HCM patients with left ventricular ejection fraction preserved (HCMpEF) compared to controls, the appearance of myocardial LGE was accompanied by a decrease in MCF and myocardial strain, and GLS and MCF could be independent predictors of the appearance of LGE in HCMpEF patients.

Serri et al. (12) showed in an ultrasound-based study that cardiomyocyte hypertrophy and myocardial fibrosis both negatively affect myocardial stress, especially longitudinal stress GLS, which has been widely used to detect subclinical cardiac insufficiency (13, 14). In our study, GLS and MCF were significantly lower in HCMpEF patients compared to controls, confirming that GLS is more sensitive to detecting subclinical myocardial damage than the conventional functional evaluation parameter LVEF. The subgroup analysis revealed that GLS was already reduced in the LGE-negative group compared to the control group, but GCS and GRS were not yet significantly different. In line with the results of the present study, our previous study based on ultrasound also suggested that impaired GLS is sensitive to detecting hidden myocardial damage in the myocardium with “normal” LGE evaluation (7). There was no difference in GCS, considering that GCS and GRS are mainly innervated by subepicardial myocardial fibers and usually show a pseudo “normal” after overcompensation in early disease, whereas GLS is innervated by subendocardial fibers and is the most sensitive index of ventricular mechanics (13–15).

Previous studies by Spartera et al. (16) and Bálint et al. (17) showed that the severity of strain injury depends to some extent on the degree of myocardial fibrosis, and our findings confirm this. Compared to LGE-negative patients, LGE-positive patients had not only lower GLS but also lower GCS and GRS, indicating that myocardial systolic function was impaired not only in the longitudinal direction but also in the circumferential and radial directions, suggesting a progression of myocardial involvement from subendocardial to subepicardial and in all directions (18). Bogarapu et al. (9), in a series of 29 patients (LGE +, *n* = 11) using 16-segment CMR feature tracking (CMR-FT), determined myocardial fibrosis in pediatric HCM. Their study novelty combined three dimensions of the entire LV to form a 16-segment LV model for the analysis of 2D and 3D regions and overall myocardial deformation and assess its relationship to LGE. However, this requires additional specialized software and isn’t included in standard PACS. Unlike our studies, we additionally assessed CMR-derived MCF in a larger sample of HCM population with left ventricular ejection fraction preserved. We found that MCF was also significantly lower in the negative group than in the control group. LVEF is usually preserved in LVH, especially in HCM, and measuring LVEF alone does not fully represent the complex process of myocardial deformation and dysfunction (19); therefore, a more sensitive marker that combines information on LV function and hypertrophy, preferably in the early stages, would be helpful. MCF is conceptually very similar to GLS, in that it is the overall contraction fraction of the left ventricle, which combines information on myocardial shortening in all regions and directions (longitudinal, radial, circumferential) and provides a direct assessment of actual myocardial contractility by combining myocardial volume, excluding the effect of chamber size (20). MCF is sensitive to detect impaired myocardial function, unlike the feature tracking analysis, which provides LV strain data that are not routinely available on standard PACS and require additional specialized software. The advantages of MCF are CMR-derived MCF with accurate and reproducible quantitative assessment does not require injectable contrast and specific post-processing software and can be easily obtained by equation calculation (21). Therefore, it does not involve cost. After correcting for confounding factors such as age, gender, and BMI, GLS, and MCF were still significantly and independently associated with the area of LGE, demonstrating that the degree of impairment of mechanical indices increases with the extent of LGE.

Another aim of this study was to explore indicators that predict myocardial LGE in HCM patients. LGE is a common manifestation of many non-ischemic cardiomyopathies, including HCM. It is the gold standard for irreversible myocardial fibrosis (4, 22), which can cause MACE events, including arrhythmias, heart failure, and sudden cardiac death. The existence of LGE indicates a relatively advanced stage of the disease. In this stage, the number of patients with renal insufficiency due to heart failure is much higher, and the need for gadolinium contrast injection for LGE testing limits its use in such patients. However, myocardial mechanics parameters can be obtained by conventional contrast-free sequences of CMR, which can predict the onset of LGE before it occurs and is important for these patients. In this study, by further constructing models and comparing them, models using basal data and GLS and MCF, respectively, had a better predictive value of LGE than models built with conventional indicators (LVEF), with a 1.613-fold increase in the risk of developing LGE for every 1% decrease in GLS in HCM patients. Our previous cardiac ultrasound-based strain study (7) found a predictive value of GLS for myocardial fibrosis in autoimmune diseases, similar to a study by Shen et al. (23). A recent study by Raucci et al. (24) also showed that GLS strongly predicts LGE emergence in Duchenne muscular dystrophy. Shimada et al. (25) and Liao et al. (26) investigated the predictive power of MCF obtained from cardiac ultrasound for HCM mortality at different time points. The aforementioned studies based on different cardiomyopathies agree with the results of the present study, confirming the potential of myocardial mechanical parameters to predict LGE occurrence.

### Clinical implications

Conventional CMR cine sequences can be more feasibly acquired and analyzed. LV strain and MCF can quantify systolic dysfunction and help better characterize subtle abnormalities in myocardial mechanics at an early stage compared to LVEF. Thus, it enhances the possibility of applying early therapeutic measures to reduce the development of myocardial cell disorders, fibrosis, and hypertrophy. Furthermore, we present a relationship between myocardial mechanical parameters and LGE, and they can be clinical markers with the potential to predict LGE. The mechanical parameters can be determined quickly and easily from standard cine CMR images without injecting contrast or post-processing software. As patients with renal failure due to low cardiac output in congestive heart failure or young adults requiring regular follow-up, mechanical parameters may reduce the need for contrast-enhanced studies in these patient groups and shorten the examination time.

### Limitation

The study has some limitations. The sample size is small, implying that future larger studies should be conducted to confirm the results. The included myocardial mechanical parameters were only compared for overall myocardial strain and excluded peak strain rate or peak exercise velocity. This was a retrospective study, so further prospective studies to investigate the role of mechanical parameters in predicting clinical outcomes such as sudden cardiac death (SCD) and heart failure are an important next step.

## Conclusion

In participants of HCM with preserved ejection fraction, the early onset of reduced left ventricular GLS and MCF in patients with HCMpEF provide new evidence for evaluating impaired myocardial systolic function. The reduction of myocardial mechanical indexes reflects the presence and extent of myocardial fibrosis; GLS and MCF may be ideal predictors for LGE.

## Data availability statement

The original contributions presented in this study are included in the article/supplementary material, further inquiries can be directed to the corresponding authors.

## Ethics statement

The studies involving human participants were reviewed and approved by The First Affiliated Hospital of Soochow University. The ethics committee waived the requirement of written informed consent for participation. Written informed consent was obtained from the individual(s) for the publication of any potentially identifiable images or data included in this article.

## Author contributions

YW and SJ contributed to the guarantor of the manuscript. XH and XX contributed to the conception, design, collection, and assembly of data. XH and XX performed the experimental studies and prepared the first draft of the manuscript, which was critically revised by YW and SJ. XL, JB, and GX contributed to the data analysis and interpretation. All authors contributed to the article and approved the submitted version.
